# State Med. Association

**Published:** 1879-03-20

**Authors:** 


### EDITORIAL AND MISCELLANEOUS.

### *STATE MEDICAL ASSOCIATION.*

The Medical Association of the State of Georgia meets in Rome on the third Monday in April next.

### *SOUTHERN MEDICAL COLLEGE.*

In response to the many inquiries in regard to this new institution we are able to state that progress is being made: a second meeting of the Board of Trustees recently held authorized the purchase of a lot, and took further action in regard to the procurement of stock, building material, etc.

Notice of the time for the election of Professors it is expected will be given at an early day.

